# Analyzing molecular signatures in preeclampsia and fetal growth restriction: Identifying key genes, pathways, and therapeutic targets for preterm birth

**DOI:** 10.3389/fmolb.2024.1384214

**Published:** 2024-04-22

**Authors:** Muhammad Bilal Azmi, Mushyeda Fatima Nasir, Uzma Asif, Mohsin Kazi, Mohammad N. Uddin, Shamim Akhtar Qureshi

**Affiliations:** ^1^ Computational Biochemistry Research Laboratory, Department of Biochemistry, Dow Medical College, Dow University of Health Sciences, Karachi, Pakistan; ^2^ Department of Biosciences, Faculty of Life Sciences, Mohammad Ali Jinnah University, Karachi, Pakistan; ^3^ Department of Biochemistry, Medicine Program, Batterjee Medical College, Jeddah, Saudi Arabia; ^4^ Department of Pharmaceutics, College of Pharmacy, King Saud University, Riyadh, Saudi Arabia; ^5^ College of Pharmacy, Mercer University, Atlanta, GA, United States; ^6^ Department of Biochemistry, University of Karachi, Karachi, Pakistan

**Keywords:** hub genes, intrauterine growth restriction, CytoHubba, flavoxate, preeclampsia, preterm birth

## Abstract

**Background:**

Intrauterine growth restriction (IUGR) and preeclampsia (PE) are intricately linked with specific maternal health conditions, exhibit shared placental abnormalities, and play pivotal roles in precipitating preterm birth (PTB) incidences. However, the molecular mechanism underlying the association between PE and IUGR has not been determined. Therefore, we aimed to analyze the data of females with PE and those with PE + IUGR to identify the key gene(s), their molecular pathways, and potential therapeutic interactions.

**Methods:**

In this study, a comprehensive relationship analysis of both PE and PE + IUGR was conducted using RNA sequence datasets. Using two datasets (GSE148241 and GSE114691), differential gene expression analysis via DESeq2 through R-programming was performed. Gene set enrichment analysis was performed using ClusterProfiler, protein‒protein interaction (PPI) networks were constructed, and cluster analyses were conducted using String and MCODE in Cytoscape. Functional enrichment analyses of the resulting subnetworks were performed using ClueGO software. The hub genes were identified under both conditions using the CytoHubba method. Finally, the most common hub protein was docked against a library of bioactive flavonoids and PTB drugs using the PyRx AutoDock tool, followed by molecular dynamic (MD) simulation analysis. Pharmacokinetic analysis was performed to determine the ADMET properties of the compounds using pkCSM.

**Results:**

We identified eight hub genes highly expressed in the case of PE, namely, PTGS2, ENG, KIT, MME, CGA, GAPDH, GPX3, and P4HA1, and the network of the PE + IUGR gene set demonstrated that nine hub genes were overexpressed, namely, PTGS2, FGF7, FGF10, IL10, SPP1, MPO, THBS1, CYBB, and PF4. PTGS2 was the most common hub gene found under both conditions (PE and PEIUGR). Moreover, the greater (−9.1 kcal/mol) molecular binding of flavoxate to PTGS2 was found to have satisfactory pharmacokinetic properties compared with those of other compounds. The flavoxate-bound PTGS2 protein complex remained stable throughout the simulation; with a ligand fit to protein, *i.e.*, a RMSD ranging from ∼2.0 to 4.0 Å and a RMSF ranging from ∼0.5 to 2.9 Å, was observed throughout the 100 ns analysis.

**Conclusion:**

The findings of this study may be useful for treating PE and IUGR in the management of PTB.

## 1 Introduction

Preterm birth (PTB), also known as premature birth, describes the birth of a baby before 37 weeks of gestation (Vogel et al., 2018). PTB can occur without an obvious cause or has a different number of causes and risk factors. These risk factors include maternal health conditions, infections, multiple pregnancies, cervical or uterine abnormalities, drug use, and medical interventions (Crump, 2020). PTB is also associated with an increased risk of various pregnancy complications, including preeclampsia (PE) and intrauterine fetal growth restriction (IUGR) (Farrelly et al., 2023). IUGR and PE have distinct links to maternal health conditions but exhibit similar placental issues and are also found to be factors contributing to PTB and stillbirth (Kajdy et al., 2023).

PE is a pregnancy-related condition characterized by the onset of high blood pressure during pregnancy and accompanied by signs of maternal organ dysfunction (Kahramanoglu et al., 2022). These signs can include the presence of new-onset proteinuria, which is an excess of protein in the urine, typically at ≥300 mg/24 h. This situation can lead to hematological issues, abnormal markers related to blood clotting and liver function, neurological problems, and indications of impaired utero-placental function, including fetal growth restriction, which generally presents after 20 weeks of gestation (LaMarca et al., 2015; Turbeville and Sasser, 2020). An adverse uterine environment, preferably PE, can trigger epigenetic alterations that have detrimental consequences on developmental adaptability with long-term effects on the fetus, such as an increased risk of morbidity that may prevail until childhood or even later adult stages (LaMarca et al., 2015; Turbeville and Sasser, 2020). PE not only has a long-term effect on the child but also on the mother, as these risks include a higher likelihood of experiencing composite adverse cardiovascular outcomes, a doubled risk of cardiovascular death, up to four times the risk of hypertension and metabolic syndrome, and more than double the risk of developing type 2 diabetes and dyslipidemia in the years following pregnancy, in addition to prematurity, growth restriction, and still birth (Lu and Hu, 2019).

IUGR refers to a condition in which a fetus does not reach its expected growth potential in the uterus (Armengaud et al., 2021). It can be caused by various factors, including placental insufficiency and maternal health conditions (Bendix et al., 2020). PTB itself can lead to growth restriction because the baby is born before it has the chance to reach full term and grow to a healthy weight. In cases where IUGR precedes PTB, restricted fetal growth may be due to placental dysfunction or other factors, making early delivery necessary to safeguard the baby’s health (Shinar et al., 2021).

Previous studies have reported the possible mechanisms underlying PTB, IUGR, and PE, establishing the role of differential gene expression (Farrelly et al., 2023). Furthermore, researchers have identified several candidate genes associated with PTB, given their involvement in diverse biological processes such as inflammation, immune response modulation, and uterine function, these biomarker genes play a crucial role in contributing to PTB. (Wadon et al., 2020; Couceiro et al., 2021; Azmi et al., 2023). Variants of these genes may increase susceptibility to PTB. PTB, PE, and IUGR are multifaceted pregnancy-related conditions with various causes, including genetic factors. Several genes and their polymorphisms have been associated with the development of these conditions; however, the specific underlying mechanisms have not been fully described (Wadon et al., 2020; Nowakowska et al., 2021).

The occurrence of PTB has many associated risks, and PE and IUGR have been reported as the main contributing consequences of PTB (Wadon et al., 2020; Couceiro et al., 2021; Nowakowska et al., 2021; Azmi et al., 2023). To comprehensively understand the underlying mechanisms of conditions such as PTB-associated PE and IUGR, it is imperative to investigate the distinct expression patterns of gene biomarkers, whether upregulated or downregulated. This approach facilitates the identification of potential inhibitors, resembling drug-like molecules, which hold promise as therapeutic interventions. In the present study, we aimed to target these Differentially Expressed Genes (DEGs) based on their expression levels and associated pathways, with the intention of designing effective drug candidates to mitigate the risks and complications associated with PTB. To achieve this goal, we proposed a meticulous analysis of DEGs by juxtaposing publicly available gene expression and RNA data from both PE and PE + IUGR placentas. Our primary objective was to pinpoint the most noteworthy gene biomarkers that could serve as future therapeutic targets, thereby laying the groundwork for PTB management strategies. We aimed to identify drug-like molecules capable of addressing this clinical challenge. Furthermore, employing Molecular Dynamics (MD) simulation analysis to scrutinize protein-ligand interactions elucidated the biochemical relevance of these interactions, thereby enhancing our experimental approaches.

## 2 Methodology

**FIGURE 1:**
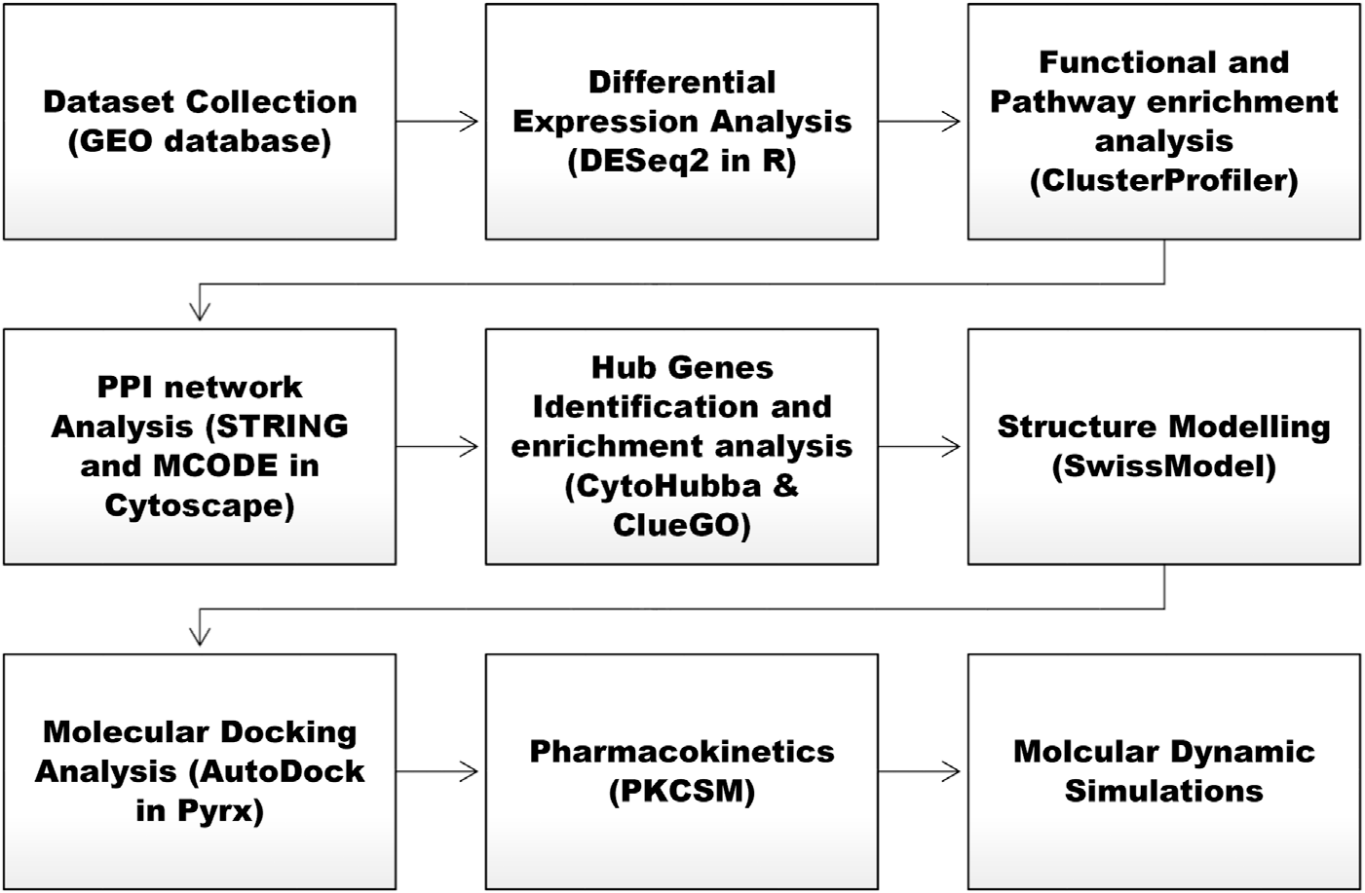
Stepwise overview of the present research study.

Figure 1 shows the stepwise methodological framework of the present research.

### 2.1 Retrieval of gene expression datasets

The RNA-sequencing datasets related to PTB were collected from the publicly available Gene Expression Omnibus (GEO) database (https://www.ncbi.nlm.nih.gov/geo/) of the National Center for Biotechnology Information (NCBI) (Barrett et al., 2012). The GEO database stands as the premier openly accessible repository housing a vast array of high-throughput molecular abundance data, with a primary emphasis on gene expression studies. Within its extensive collection, it encompasses a diverse spectrum of data types, spanning from traditional microarray-based evaluations of mRNA, genomic DNA, and protein levels to non-array methodologies like serial analysis of gene expression (SAGE) and mass spectrometry for comprehensive proteomic analyses. In our investigation, we procured two distinct RNA sequence datasets specifically focusing on females afflicted with preterm birth (PTB), with one dataset including pertinent data on preeclampsia (PE) status, sourced from the GEO accession GSE148241 (Yang et al., 2020). and the other with PE + IUGR (preeclampsia and intrauterine fetal growth restriction) from the GEO accession GSE114691 (Awamleh et al., 2019). The first RNA sequence dataset, GSE148241, encompassed RNA-seq data obtained from a total of 41 placenta tissue samples. Which included the placentae of 9 individuals diagnosed with early-onset severe preeclampsia (EOSE), in comparison with those of 32 healthy controls, utilizing the Illumina HiSeq 2,500 platform. Moving to the second RNA sequence dataset, GSE114691, high-throughput expression profiling was conducted using the Illumina HiSeq 2000 platform. This dataset, scrutinized in our investigation, entailed RNA-seq data from 79 samples. Of our main interest were the samples from intrauterine growth restriction (IUGR) and early-onset preeclampsia (PE), the pregnancies complicated by both conditions. The placental specimens sourced from four distinct patient cohorts, including PE + IUGR. 20 samples with PE_IUGR were included in the analysis, while a control group comprising 21 subjects served as reference.

### 2.2 Differential expression analysis of genes

To determine the differential expression of the genes in the above datasets, differential expression analysis was performed using the DESeq2 library in the R programming language (Love et al., 2014). The library operates by augmenting the robustness and comprehensibility of differential expression estimations via the implementation of shrinkage estimation techniques for dispersions and fold changes. In this analytical framework, differential expression analysis was governed by stringent criteria, requiring a minimum log2 fold change threshold of >1 for upregulated genes and <-1 for downregulated genes. Additionally, a significance threshold of *p*-value >0.05 was uniformly applied across both the selected Pre-Eclampsia (PE) and PE + Intrauterine Growth Restriction (IUGR) datasets, ensuring rigorous statistical scrutiny and reproducibility in the identified gene expression alterations.

### 2.3 Gene functional and pathway enrichment analysis

Functional enrichment analysis of the set of DEGs was performed using the gseGO and gseKEGG methods of the clusterprofiler in the R programming language. This approach allows for the extraction of semantic pathways and Gene Ontology (GO) terms in which the list of DEGs is found to be enriched. The clusterProfiler was used to construct the GO and KEGG (Kyoto Encyclopedia of Genes and Genomes) datasets for multiple model organisms. The primary purpose of this approach was to facilitate the comparison of functional profiles across distinct conditions at a single level, such as various treatment groups (Wu et al., 2021). The parameters were set as follows: minimum gene set size, 3; maximum set size, 800; and *p-value* < 0.05.

### 2.4 Protein–protein interaction (PPI) network analysis

We particularly focused on the upregulated DEGs from both datasets side by side; these DEGs were further analyzed to reveal the PPI network using STRING (Von Mering et al., 2003) and the plug-in in Cytoscape 3.9.1 (Kohl et al., 2011; Saito et al., 2012). Cluster (subnetwork) analysis was performed using MCODE, which allows for the recognition of local subnetworks from the larger network that have the most significance based upon the higher score and strength of the nodes and edges connecting them. These gene clusters were analyzed to closely study the processes and pathways in which the genes were enriched using ClueGO (Bindea et al., 2009) in Cytoscape.

### 2.5 Identification of hub genes and functional enrichment

Hub genes were defined as genes with a high correlation in candidate modules. These genes were basically defined by the number of associations (*i.e.,* node degree); highly associated genes were denoted as hub genes. The hub genes were identified using CytoHubba in Cytoscape (Chin et al., 2014). This approach employs twelve distinct methods, including MCC, EPC, MNC, DMNC, eccentricity, radiality, stress, betweenness, closeness, clustering coefficient, bottleneck, and degree, to identify key genes within biological networks. These methods assess various network properties such as connectivity, centrality, and structural importance to pinpoint genes that play pivotal roles in regulating network behavior. Genes that were identified by five or more of these methods were classified as hub genes, signifying their significance in the network topology and their potential importance in biological processes under investigation.

### 2.6 Structural prediction of hub proteins, library compilation of natural compounds and PTB drugs and molecular docking

The 3D model of the identified Hub proteins was investigated with the aid of protein-BLAST (Basic Local Alignment Search Tool) for identifying the structural similarity of the desired proteins in the PDB repository (Stover and Cavalcanti, 2017). In the context of incomplete structural data available in the PDB, indicating a gap in the structure, we employed an alternative homology modeling technique to construct a homology model for the Hub protein common to both datasets. To evaluate the completeness or maximum coverage of the 3D model, we utilized the SWISS-MODEL web server for homology modeling. (Robin et al., 2024). Following the generation of the respective 3D homology models, structural validation was conducted using verify-3D, ERRAT, and PROCHECK-based assessments in Saves6.0.(Dym et al., 2012).

In addition, a 3D compound library comprising bioactive flavonoids reported from various sources and from various classes was retrieved from the PubChem compound database (https://pubchem.ncbi.nlm.nih.gov). The 3D structures of all the reported compounds were retrieved, and following a visual examination of each 3D compound, the structures were initially downloaded in SDF (structure data format) and subsequently translated to PDB (protein database) using Open Babel software (Yoshikawa and Hutchison, 2019). All the 3D compounds were subjected to *Pfizer’s rule* of five for evaluating drug-likeness criteria (Tinworth and Young, 2020). Following the evaluation, only 3D bioactive flavonoids that satisfied all five of *Pfizer’s Rule* of Five physicochemical requirements were chosen because any one infraction was treated as a failure or an elimination aspect. Only eligible compounds were included and further subjected to docking analysis. According to the criteria previously reported by Azmi et al., in 2023, we selected different PTB drugs for reference and comparison (Azmi et al., 2023).

PyRx is a platform that automates and integrates various tools for molecular docking simulations, streamlining the process of virtual screening for small-molecule library screening. In our study, we utilized PyRx to prepare input files for docking by converting them to the PDBQT file format compatible with AutoDock Vina. After comprehensive stereo-chemical investigations of the selected 3D model, the protein was docked to a library of 61 bioactive flavonoid compounds and 5 PTB drugs in PyRx (Ounthaisong and Tangyuenyongwatana, 2017). The grid parameters used for this analysis were X: 20.0265, Y: 35.5676, and Z: 61.2142. The complexes with the most favorable binding energies and RMSD (root mean square deviation) values equal to zero were selected for further pharmacokinetic studies using pkCSM (Pires et al., 2015).

### 2.7 Molecular dynamic simulation and MM-GBSA calculations

Desmond, a software application from Schrödinger LLC, was used to run 100 nanoseconds of molecular dynamics (MD) simulations. For this purpose, rigid binding of the chosen chemical compound against the target protein was calculated via receptor–ligand docking via MD simulation (Bowers et al., 2006; Ferreira et al., 2015). By using Newton’s classical equation of motion, MD simulation investigations were carried out to predict the ligand binding status in the physiological environment (Hildebrand et al., 2019; Rasheed et al., 2021). Using Maestro’s Protein Preparation Wizard, the chosen proteins and ligands were optimized and reduced. There was no longer any steric conflict, poor contact, or distorted geometry. The systems were constructed using the System Builder tool, and TIP3P (Intermolecular Interaction Potential 3 Points Transferable), an orthorhombic box with the OPLS_2005 force field, was utilized as the solvent model (Shivakumar et al., 2010; Malik et al., 2023). Throughout the simulation period, a 300 K temperature and 1 atm pressure were utilized to simulate physiological conditions, while counterions were added to neutralize the models, and 0.15 M sodium chloride was added. Trajectories were saved after every 100 picoseconds (ps) for inspection, and the stability of the protein–ligand complex was determined by measuring the root mean square deviation (RMSD) over time. Using the Bio3D package of R, principal component analysis (PCA) and a dynamic cross-correlation matrix (DCCM) were examined; however, PCA and DCCM were computed using an R language script (Malik et al., 2023; Ullah et al., 2023).

In generalized molecular mechanics, during MD simulations of the above protein‒ligand complex, the Born surface area (MM-GBSA) module of Prime was utilized to calculate the binding free energy (Gbind) of the docked complex. The binding free energy was calculated using the OPLS 2005 force field, VSGB solvent model and rotamer search methods. After the MD run, the MD trajectory frames were selected at intervals of 10 ns (Ullah et al., 2023).

$$\Delta G_{\text{bind}}=G_{\text{complex}}\;–\;\left(G_{\text{protein}}+G_{\text{ligand}}\right)$$

In this equation, ΔG_bind_ = binding free energy, G_complex_ = free energy of the complex, G_protein_ = free energy of the target protein, and G_ligand_ = free energy of the ligand.

## 3 Results

### 3.1 Datasets associated with differentially expressed genes

**FIGURE 2:**
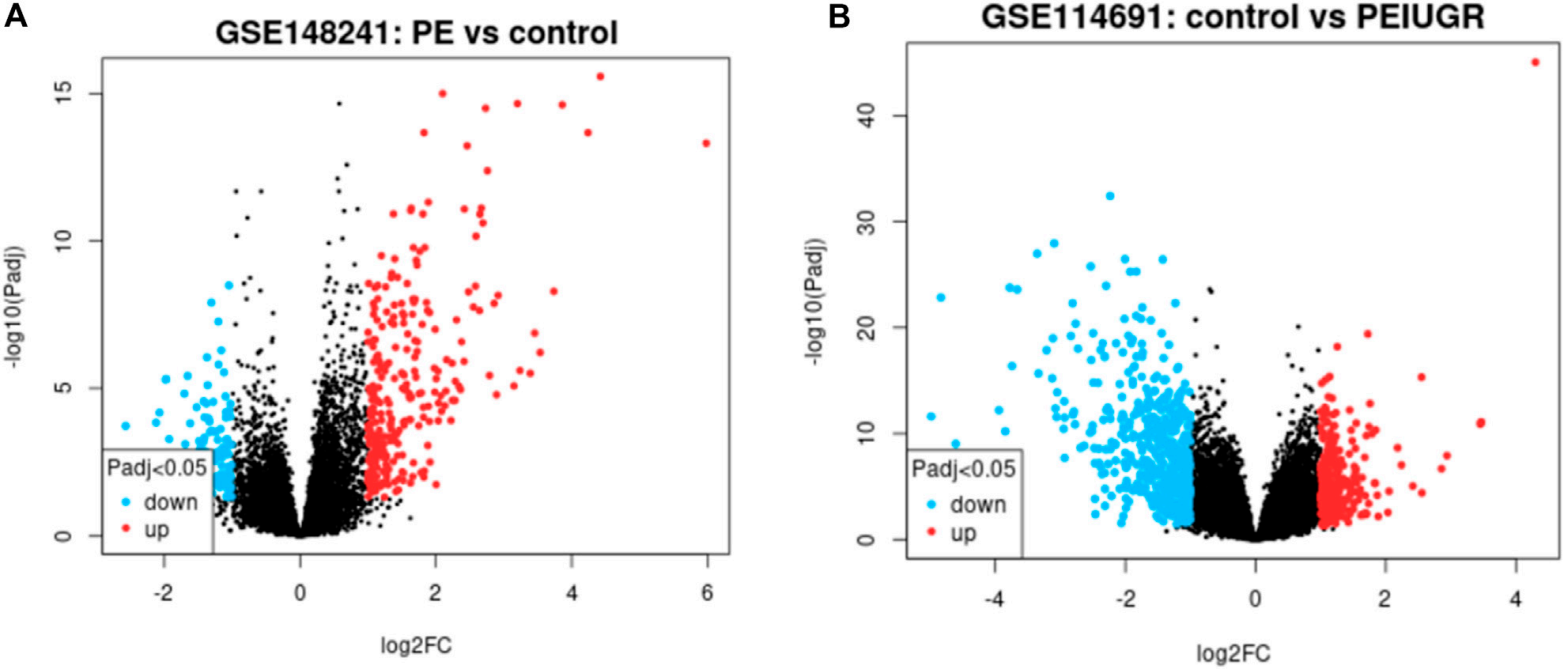
Volcano plot showing the PE dataset **(A)** and the PE + IUGR dataset **(B)**. The thresholds of ≤ −1 and ≥1 upon log_2_(fold change) and *p-value < 0.05* were applied; red indicates upregulated genes, while blue indicates downregulated genes, and black dots represent normally expressed genes in both dataset plots.

**FIGURE 3:**
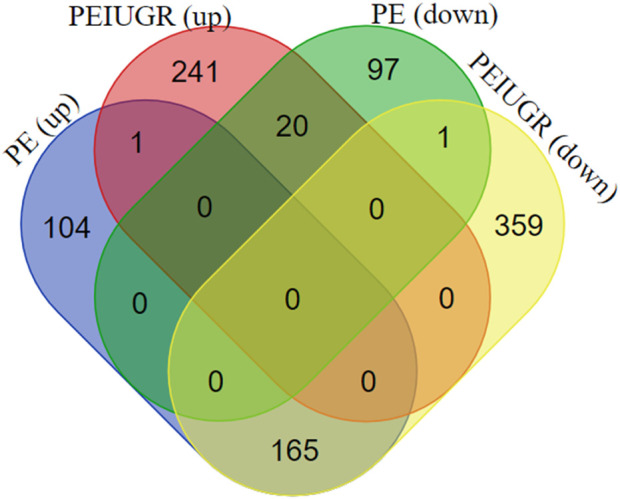
Venn diagram of DEGs (upregulated and downregulated) in the PE and PE + IUGR datasets. PE (up) and PE + IUGR (up) had 1 gene in common, PE (up) and PE + IUGR (down) had 165, PE (down) and PE + IUGR (up) had 20, and PE (down) and PE + IUGR (down) had 1 gene in intersection.

**FIGURE 4:**
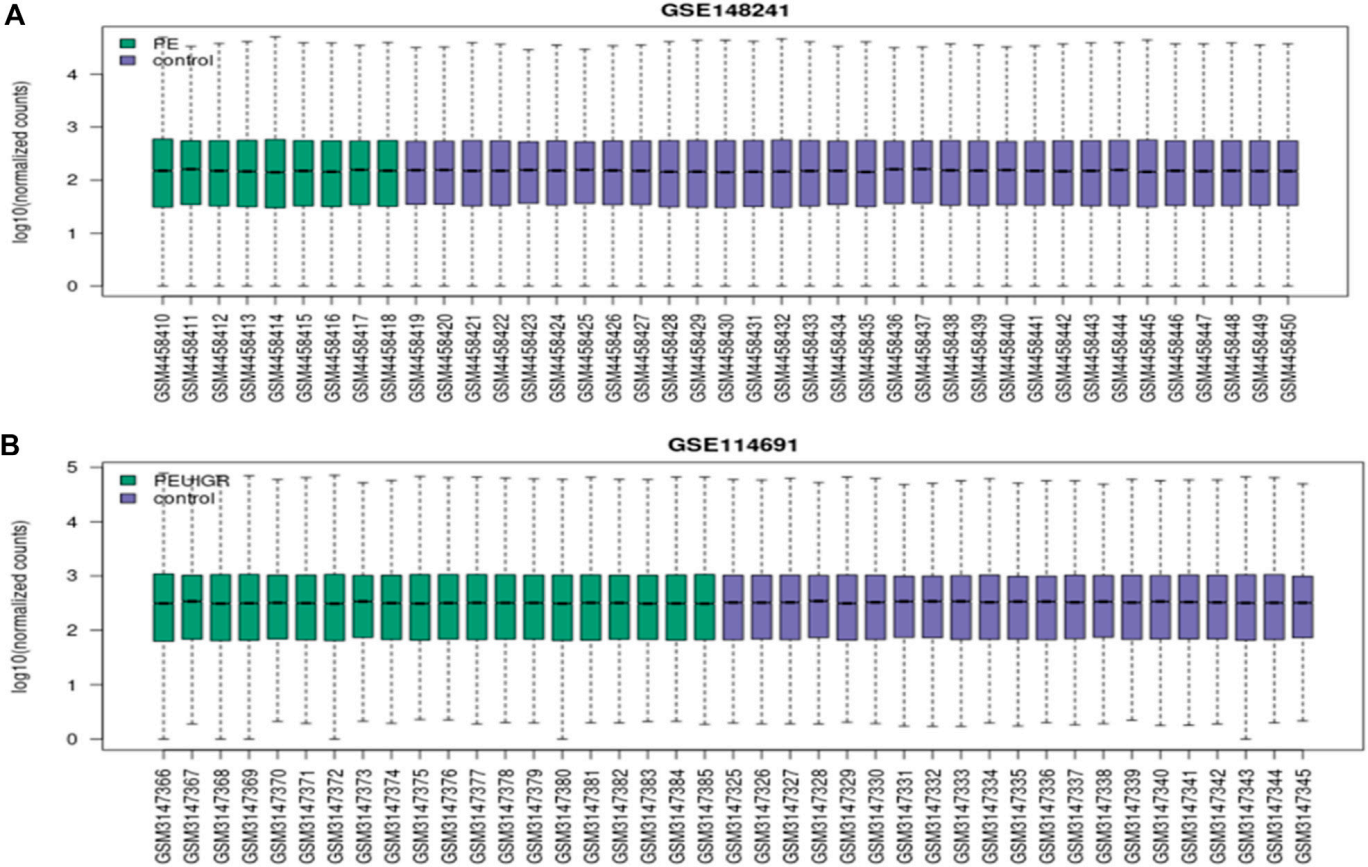
Boxplots of the PE dataset **(A)** and the PE + IUGR dataset **(B)**; the green color represents the condition (PE or PE + IUGR) while the blue color is for representing the control group. X-axis represents the accession of each individual sample while the log10 of normalized counts is represented on the y-axis.

The thresholds of < −1 and >1 upon log2(fold change) and *p*-value <0.05 yielded 270 upregulated genes and 119 downregulated genes, respectively, in the case of PE. Some of the top genes included FSTL3, SPAG4, FLT1, LOC105370135, TMEM45A, HTRA4, SLC6A8, and LEP, all of which were overexpressed (Table 1; Supplementary Table S1). However, under the PE + IUGR condition, 262 genes were upregulated, 525 were downregulated, and the top genes included SLAMF1, QPCT, NTRK2, PNCK, ENG, HEXB, MYO7B, LOC102723566, PROCR, CLDN9 and FSTL3 (Table 2; Supplementary Table S2). We also found that none of the genes were upregulated; these genes were common in both datasets, except for PTGS2, and were highly expressed in both datasets. CA1 was the only gene commonly downregulated in both datasets. Interestingly, 20 of the genes whose expression was suppressed in the PE dataset were overexpressed in the PE + IUGR dataset; these genes included RALYL, LOC101928957, OGN, MYO1A, CLDN16, ZNF385B, LOC105377546, LOC107986064, SLAMF1, LOC107985906, RNASE11, SELE, LRRN3, NRAD1, EWSAT1, TRIL, SCN4B, SPP1, ITIH3, and CNTN6 (Figure 2; Figure 3). Figures 4A, B indicates the comparative status (with relation to controls) of both datasets (i.e., PE and PE + IUGR)

**TABLE 1:** Top 20 DEGs in preeclampsia (PE) patients.

Gene Entrez ID	p-adj	*p-value*	log_2_ Fold change	Base-mean	Symbol	Direction of expression
10,272	2.63E-16	1.38E-20	4.423635	8647.75	FSTL3	upregulated
6,676	1.01E-15	1.06E-19	2.100103	117.68	SPAG4	upregulated
2,321	2.20E-15	3.97E-19	3.201478	37085.81	FLT1	upregulated
105,370,135	2.41E-15	6.29E-19	3.861228	27.68	LOC105370135	upregulated
55,076	3.17E-15	9.95E-19	2.732278	145.68	TMEM45A	upregulated
203,100	2.13E-14	7.84E-18	4.240723	5046.19	HTRA4	upregulated
6,535	2.13E-14	8.92E-18	1.825831	1358.9	SLC6A8	upregulated
3,952	4.87E-14	2.29E-17	5.981728	6561.98	LEP	upregulated
100,506,211	5.96E-14	3.12E-17	2.459479	114.45	MIR210HG	upregulated
353,149	4.15E-13	2.61E-16	2.759095	40.75	TBC1D26	upregulated
23,328	4.88E-12	4.34E-15	1.888869	1407.63	SASH1	upregulated
4,648	7.76E-12	7.60E-15	2.669548	543.96	MYO7B	upregulated
2,317	7.76E-12	7.71E-15	1.637079	2843.13	FLNB	upregulated
139,728	8.32E-12	8.70E-15	2.417453	62.51	PNCK	upregulated
105,377,105	9.36E-12	1.08E-14	1.628198	209.7	FLNB-AS1	upregulated
2,630	1.21E-11	1.58E-14	1.374037	766.98	GBAP1	upregulated
102,723,566	1.21E-11	1.58E-14	1.809025	1963.99	LOC102723566	upregulated
151,242	1.23E-11	1.67E-14	2.648589	23.23	PPP1R1C	upregulated
5918	2.46E-11	3.61E-14	2.693015	48.18	RARRES1	upregulated
105,379,133	6.92E-11	1.09E-13	2.589534	38.39	LOC105379133	upregulated

**TABLE 2:** Top 20 DEGs in patients with preeclampsia (PE) and intrauterine growth restrictions (PE + IUGR).

Gene Entrez ID	Padj	*p-value*	log_2_ Fold change	Base-mean	Symbol	Direction of expression
6,504	8.78E-46	4.63E-50	4.299709	155.3	SLAMF1	upregulated
25,797	3.73E-33	3.94E-37	−2.2361	207.55	QPCT	downregulated
4,915	1.12E-28	1.77E-32	−3.09433	335.27	NTRK2	downregulated
139,728	1.05E-27	2.22E-31	−3.35737	207.1	PNCK	downregulated
2022	3.44E-27	9.07E-31	−2.00732	25,391.99	ENG	downregulated
3,074	3.65E-27	1.16E-30	−1.4259	15676.49	HEXB	downregulated
4,648	1.67E-26	6.15E-30	−2.53467	938.68	MYO7B	downregulated
102,723,566	5.16E-26	2.18E-29	−1.83316	1483.44	LOC102723566	downregulated
10,544	5.30E-26	2.52E-29	−1.92722	3,249.11	PROCR	downregulated
9,080	1.15E-24	6.06E-28	−2.29867	119.34	CLDN9	downregulated
10,272	1.68E-24	9.75E-28	−3.7777	28457.66	FSTL3	downregulated
203,100	2.51E-24	1.72E-27	−3.66321	9683.23	HTRA4	downregulated
105,377,068	1.46E-23	1.16E-26	−4.83591	119.24	LINC02009	downregulated
8076	4.97E-23	4.20E-26	−1.23381	5324.42	MFAP5	downregulated
1474	5.01E-23	4.50E-26	−2.81134	171.4	CST6	downregulated
219,539	1.25E-22	1.19E-25	−1.74071	89.82	YPEL4	downregulated
387,882	7.75E-22	7.77E-25	−1.8341	335.12	C12orf75	downregulated
2,630	1.46E-21	1.54E-24	−1.75978	3849.63	GBAP1	downregulated
84,960	1.50E-21	1.67E-24	−2.01514	374.88	CCDC183	downregulated
23,328	2.08E-21	2.53E-24	−1.61066	3723.47	SASH1	downregulated

### 3.2 Gene set enrichment analysis

**FIGURE 5:**
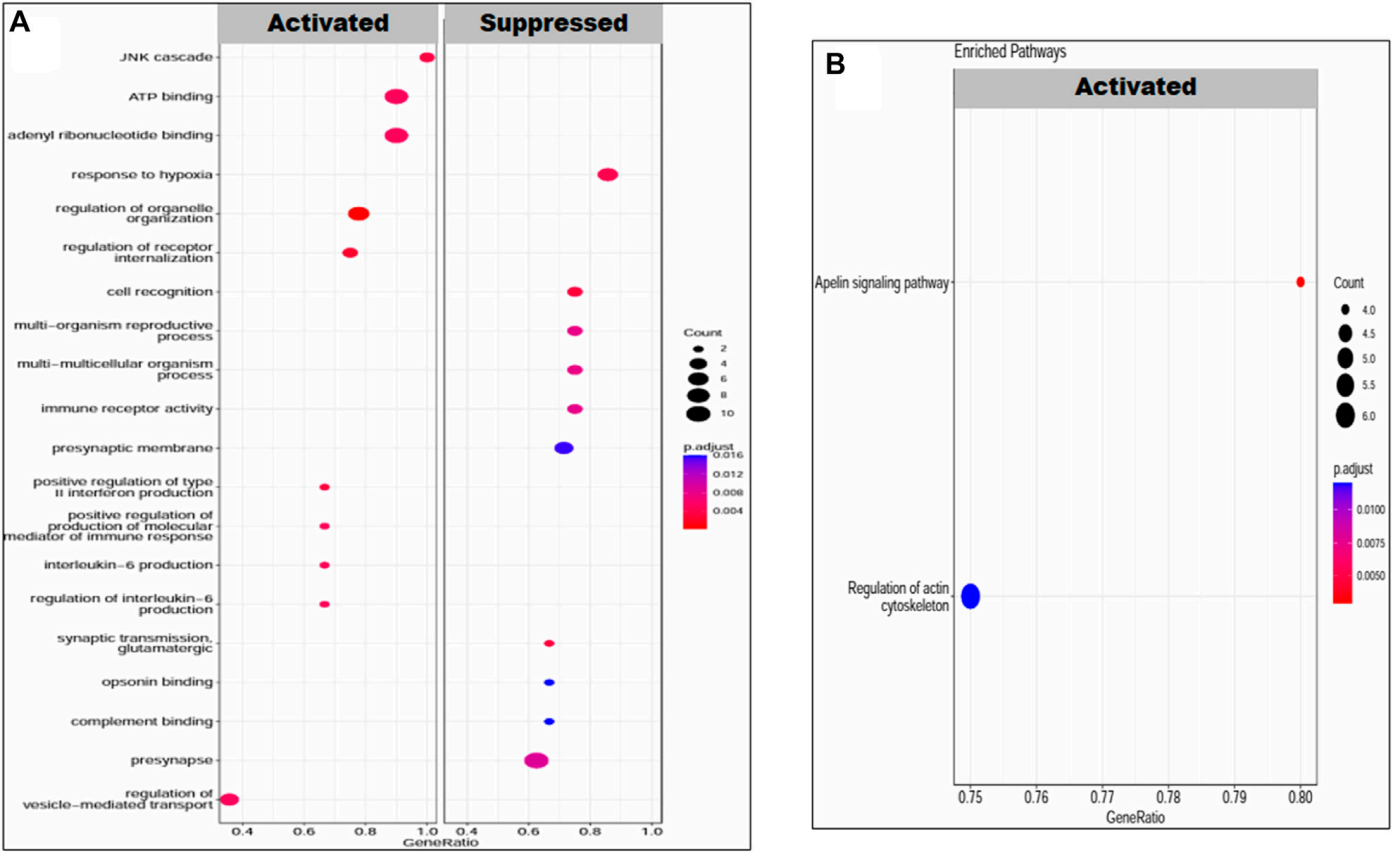
Enrichment of DEGs in PE patients. **(A)** GO terms: biological processes, molecular functions and cellular components and **(B)** KEGG pathways. The transition in color from red to blue represents the *log10 p-value*, while the size of the dot is proportional to the number of genes enriched in that particular process or pathway.

Gene set enrichment analysis revealed the molecular functions, biological processes, cellular components, and pathways in which our set of DEGs was enriched under both conditions, that is, placenta with PE and PE + IUGR. The set of PE + IUGR DEGs was mostly enriched in GO terms such as response to hypoxia, regulation of organelle organization, regulation of receptor internalization, cell recognition, synaptic transmission, glutamatergic, positive regulation of type II interferon production, and the JNK cascade and in KEGG pathways such as the apelin signaling pathway and regulation of the actin cytoskeleton. Specifically, enrichment in terms related to “response to hypoxia” could indicate that these DEGs are involved in the cellular response to reduced oxygen levels, which is often associated with complications during pregnancy, including PTB and IUGR. Furthermore, terms such as “regulation of organelle organization” and “regulation of receptor internalization” may imply that these DEGs are involved in maintaining cellular structure and signaling processes, which are critical for fetal development. Additionally, the presence of terms related to the immune response (“positive regulation of type II interferon production”) and cellular signaling pathways (“JNK cascade”) suggested potential mechanisms underlying PTB and IUGR, possibly related to immune regulation and cell signaling abnormalities (Figure 5).

**FIGURE 6:**
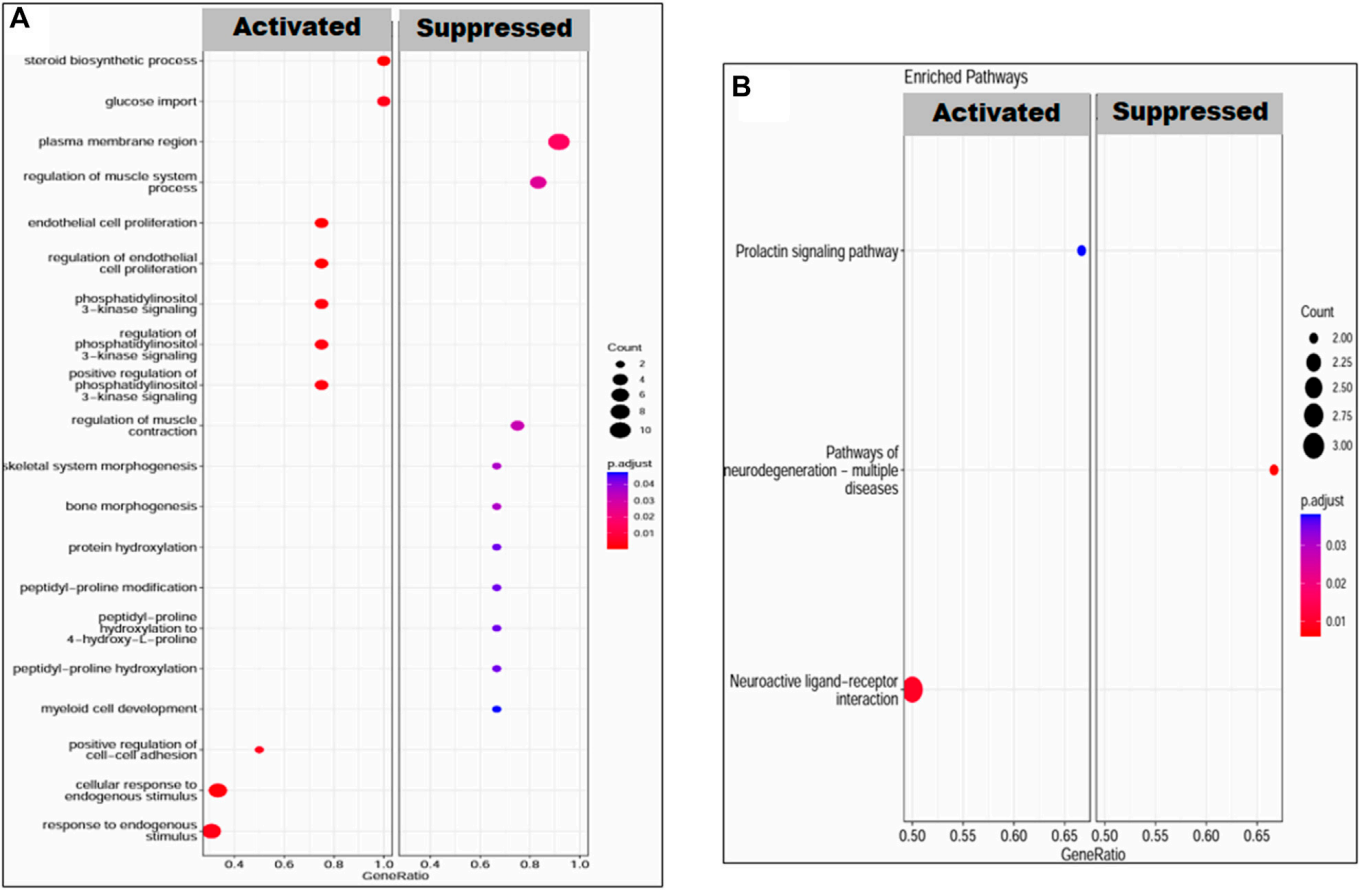
Enrichment of DEGs in PEIUGR patients. **(A)** GO terms: biological processes, molecular functions and cellular components and **(B)** KEGG pathways. The transition in color from red to blue represents the log10 *p-value*, while the size of the dot is proportional to the number of genes enriched in that particular process or pathway.

The upregulated KEGG pathways in the case of PE DEGs were related to neuroactive and prolactin signaling pathways, with a set of 6 and 3 genes involved, respectively. The neurodegenerative pathway was downregulated, with a net enrichment score (NES) of −1.82. The top enriched GO terms were steroid biosynthetic process, regulation of endothelial cell proliferation, cellular response to endogenous stimulus, and phosphatidylinositol 3-kinase signaling (Figure 6).

### 3.3 PPI network and subcluster analysis reveal hub genes

The construction of a PPI network for further analysis of DEGs serves as a highly significant step, as it shows how the proteins encoded by the set of DEGs interact with each other to carry out cellular processes and how disturbing their expression can be problematic in maintaining the balance. CytoHubba analysis of the STRING network of proteins from both datasets helped unveil the hub genes based on the 12 methods. We identified 8 hub genes involved in PE, namely, PTGS2, ENG, KIT, MME, CGA, GAPDH, GPX3 and P4HA1, while the network of the PE + IUGR gene set included 9 hub genes, namely, PTGS2, FGF7, FGF10, IL10, SPP1, MPO, THBS1, CYBB and PF4 (Table 3). MCODE revealed 4 clusters (cluster 1 with a score >3.50) for the PE condition (Table 4) and 7 cluster modules (4 clusters with a score >3.50) for the PE + IUGR condition (Table 4). These clusters help analyze the local subnetworks from the larger networks so that nodes of the highest significance can be identified.

**TABLE 3:** Hub genes identified in the PE and PE + IUGR groups based on the number of occurrences according to the CytoHubba method.

Condition	Hub gene	Number of occurrences in CytoHubba methods
PE	KIT	10
PE	GAPDH	10
PE	PTGS2	8
PE	ENG	8
PE	CGA	6
PE	MME	6
PE	GPX3	6
PE	P4HA1	6
PE + IUGR	PTGS2	9
PE + IUGR	SPP1	9
PE + IUGR	1L10	8
PE + IUGR	MPO	7
PE + IUGR	PF4	6
PE + IUGR	FGF10	6
PE + IUGR	THBS1	6
PE + IUGR	FGF7	5
PE + IUGR	CYBB	5

**TABLE 4:** The strength of each subnetwork (cluster) within the PPI network in PE and PE + IUGR represented by the number of nodes and edges.

Condition	Cluster number	Nodes	Edges	Score
PE	1	5	10	5.00
PE	2	4	5	3.33
PE	3	10	14	3.11
PE	4	3	3	3.00
PE + IUGR	1	13	53	8.83
PE + IUGR	2	26	14	5.6
PE + IUGR	3	11	25	5.00
PE + IUGR	4	12	27	4.909
PE + IUGR	5	3	3	3.00
PE + IUGR	6	3	3	3.00
PE + IUGR	7	3	3	3.00

### 3.4 Functional set enrichment of protein cluster and hub gene interactions

Cluster 1 from the PE dataset included 10 edges and 5 nodes and was particularly significant due to its functional enrichment related to the interaction between chorionic gonadotropin alpha subunit (CGA) and chorionic gonadotropin beta subunit 3 (CGB3) to form human chorionic gonadotropin (hCG) Figures 71A, 1B. When analyzing the significant clusters from the PE + IUGR dataset, we found that cluster 1 proteins were involved in processes such as negative regulation of the response to wounding, the fibroblast growth factor receptor signaling pathway, positive regulation of keratinocyte migration, and lung fibrosis [Figures 72A, 2B]. Cluster 2, containing 6 nodes and 14 edges, was enriched in the sodium channel complex, and Ankyrins linked voltage-gated Na+ and K+ channels to spectrin and L1 [Figures 73A, 3B]. Cluster 3 included 11 nodes, and 25 were associated with processes related to defense mechanisms, such as neutrophil chemotaxis and lymphocyte chemotaxis [Figures 74A, 4B]. Cluster 4 was linked to cellular processes, such as miRNA involvement in the immune response in sepsis, SARS-CoV-2 innate immunity evasion, and negative regulation of T-cell proliferation (Figures 75A, 5B)). The geneMANIA network of the hub genes from both conditions revealed local interactions with other genes in a functional network based on co-expression, shared protein domains, co-localization, and genetic interactions (Figure 8).

**FIGURE 7:**
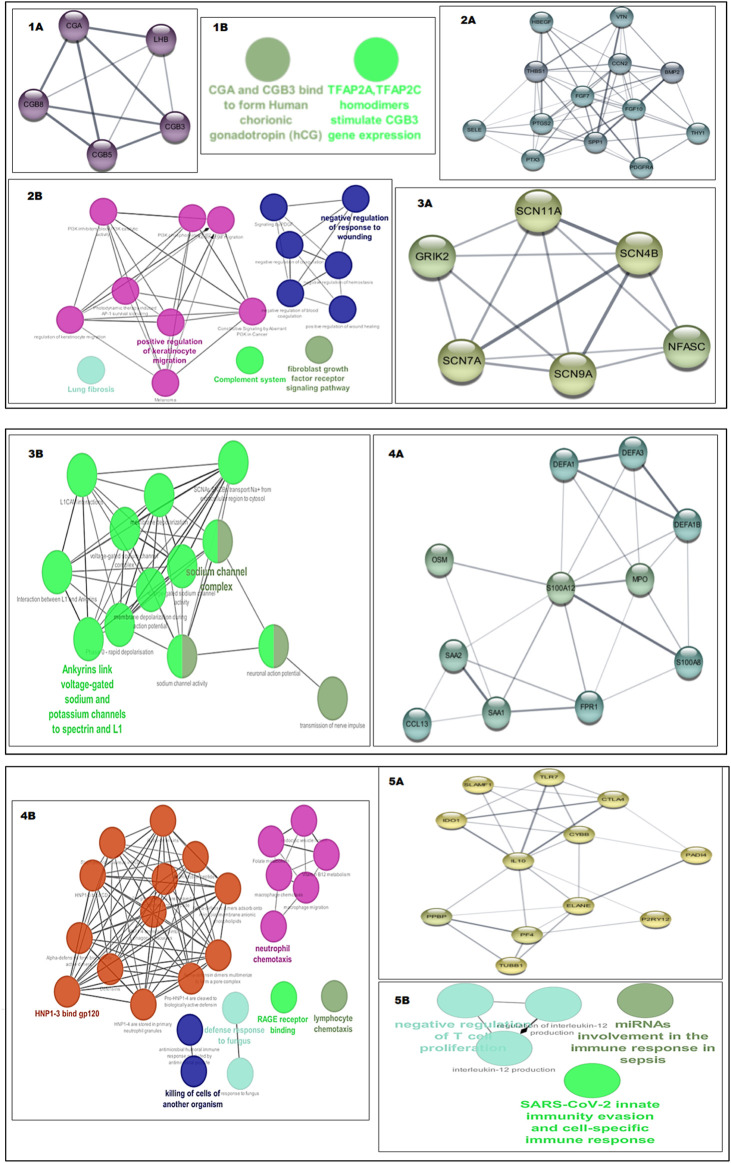
Shows the sub networks of significance and their functional enrichment, i.e., GO terms and KEGG pathways analyzed using ClueGo, the color transition of gene network from yellow to violet is based upon the score of network, a more violet color shows more network strength **(1A)** Cluster1 of PE **(1B)** Functional enrichment of cluster1 of PE, **(2A)** Cluster1 of PE + IUGR **(2B)** Functional enrichment of cluster1 of PE + IUGR, **(3A)** Cluster2 of PE + IUGR **(3B)** Functional enrichment of cluster2 of PE + IUGR, **(4A)** Cluster3 of PE + IUGR **(4B)** Functional enrichment of cluster3 of PE + IUGR, **(5A)** Cluster4 of PE + IUGR **(5B)** Functional enrichment of cluster4 of PE + IUGR.

**FIGURE 8:**
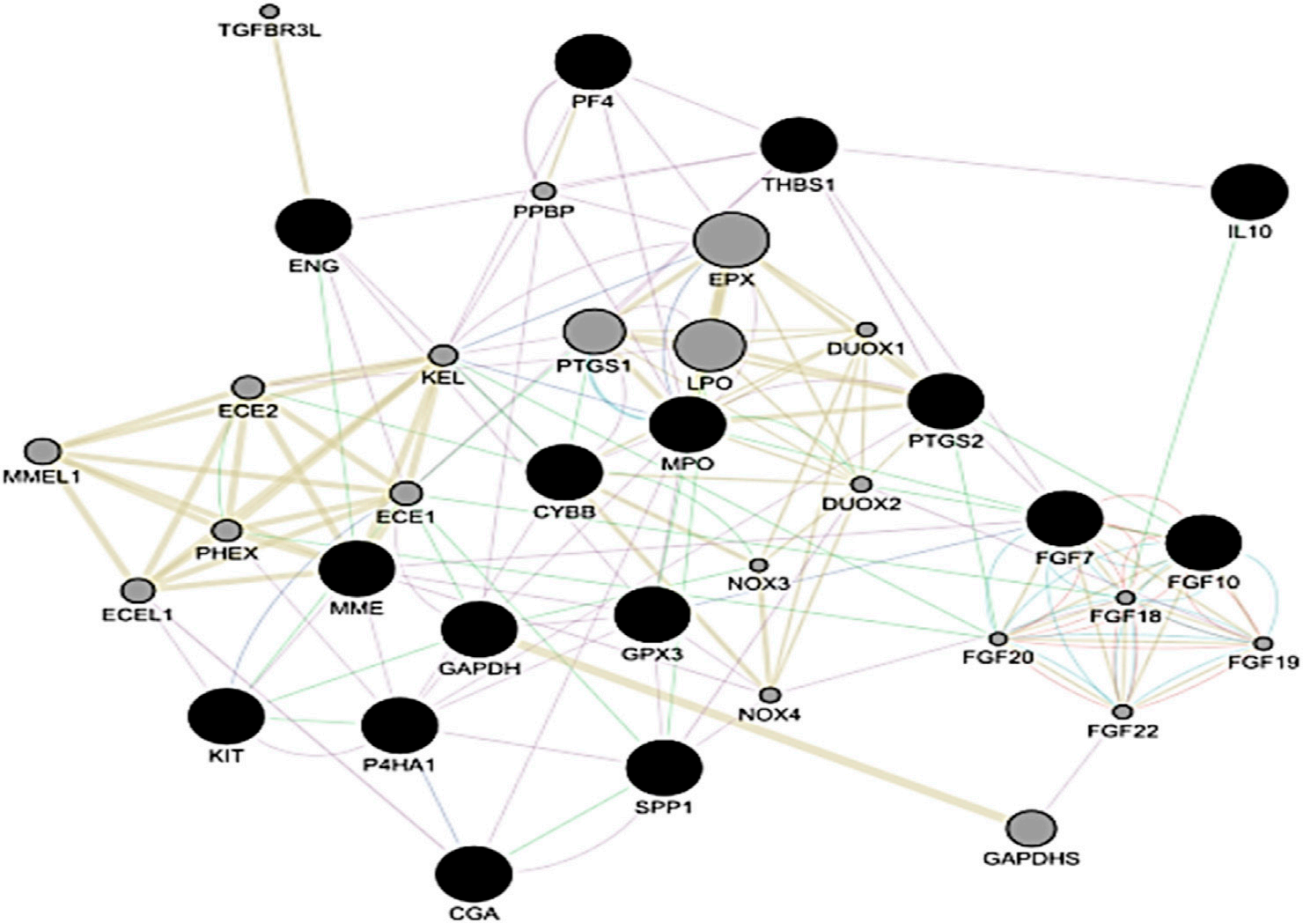
Gene–gene interaction network of hub genes based upon co-expression, shared protein domains, co-localization and genetic interaction from both the PE and PE + IUGR datasets. The black nodes represent the Hub genes.

### 3.5 Homology modeling and structural validation of the PTGS2 protein

PTGS2 was found to be common between both conditions as a highly expressed gene and was among the top hub genes, suggesting that it can be used as a promising target in combating both conditions related to PTB, that is, PE and PE + IUGR. The prostaglandin G/H synthase 2 precursor (PTGS2) consists of 604 amino acid residues. The protein 3D structure is available in the Protein Data Bank under accession number 5f19; however, to fill these gaps, five 3D homology models were generated (Table 5). The selected 3D model of PTGS2 had 91.5% of the residues lying in the most favored regions according to the Ramachandran plot. Verification of 3D showed that 76.04% of the residues had an average 3D-1D score >0.1, while ERRAT showed a 3D model quality score of 95.94%.

**TABLE 5:** Basic Local Alignment Search Tool (BLAST) analysis of the 3D homology models generated using the SWISS-MODEL for the PTGS2 protein.

Model	PDB accession (A)	Name	Method with structure resolution	Sequence Identity (%)	Query coverage (%)	Oligo-state	Global model quality Estimate (GMQE)
1	5f19.1	The Crystal Structure of Aspirin Acetylated Human Cyclooxygenase-2	X-ray, 2.04 Å	100	91	Homodimer	0.88
2	5ikr.1	The Structure of Mefenamic Acid Bound to Human Cyclooxygenase-2	X-ray, 2.34 Å	100	91	Homodimer	0.88
3	5kir.1	The Structure of Vioxx Bound to Human COX-2	X-ray, 2.70 Å	99.82	91	Homodimer	0.88
4	5f1a.1	The Crystal Structure of Salicylate Bound to Human Cyclooxygenase-2	X-ray, 2.38 Å	100	91	Homodimer	0.88
5	P35354.1	AlphaFold DB model of PGH2_HUMAN	AlphaFold v2	100	100	Monomer	0.93

### 3.6 Molecular docking and MD simulation of the PTGS2 protein with flavonoids

**FIGURE 9:**
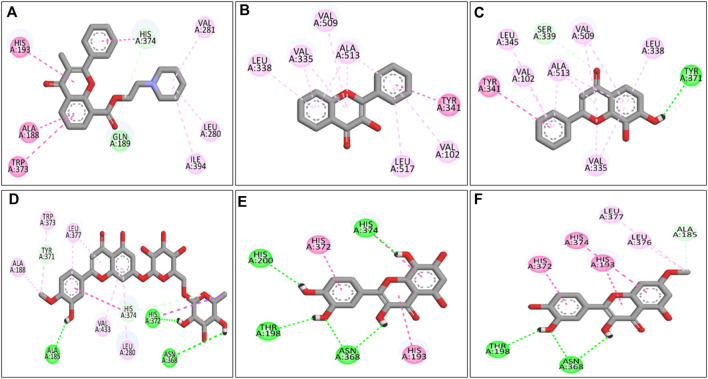
2-D ligand‒receptor interactions of **(A)** flavoxate, **(B)** 3-hydroxyflavone, **(C)** 7,8-dihydroxyflavone, **(D)** diosmin, **(E)** gossypetin, and **(F)** rhamnetin. Dark green shows H-bonds, light green shows C-H bonds, dark pink shows Pi-Pi bonds, and light pink shows alkyl bonds.

Molecular docking analysis of the flavonoid compounds and PTB drugs revealed significant binding affinities for the PTGS2 protein. Overall, 59 out of the 66 flavonoid compounds exhibited good binding affinities in the range of −7.5 to −9.1 kcal/mol, with a root mean square deviation (RMSD) of zero (Supplementary Table S3). Similarly, the PTB drugs allylestrenol, ritodrine, and retinosban also showed good affinities within the favored range, while terbutaline and hydroxyprogesterone caproate had binding energies higher than that of the target protein PTGS2 (Supplementary Table S3). The flavonoid compounds with the most favorable binding energies were flavoxate (−9.1 kcal/mol), disomin (−9 kcal/mol), 7,8-dihydroxyflavone (−8.9 kcal/mol), 3-hydroxyflavone (−8.9 kcal/mol), gossypetin (−8.8 kcal/mol) and rhamnetin (−8.8 kcal/mol). Some of the common interacting residues in the target protein were HIS374, ALA188, TRP373, VAL102, LEU517, ALA513, and HIS372 (Table 6). In addition, the PTB drugs, such as Ritodrine (−8.1 kcal/mol), Hydroxyprogesterone caproate (−6.7 kcal/mol), Retosiban (−8.1 kcal/mol), Terbutaline (−6.8 kcal/mol) and Allylestrenol (−7.8 kcal/mol), had less favorable binding energies (Supplementary Table S3; Figure 9).

**TABLE 6:** Five top-ranked docked protein‒ligand interactions with the target protein ‘PTGS2’ and its molecular interactions.

Ligand ID	Ligand name	Binding Affinity (kcal/mol)	Interacting residues of PTGS2	Residues forming C-H bond	Residues forming H-bonds
3,354	Flavoxate	−9.1	HIS374, VAL281, LEU280, ILE394, GLN189, ALA188, TRP373, HIS193	HIS374, GLN189	-
11,349	3-Hydroxyflavone	−8.9	VAL102, VAL335, LEU383, TYR341, VAL509, ALA513, LEU517	None	-
1880	7,8-dihydroxyflavone	−8.9	VAL102, ALA513, VAL509, LEU338, TYR371, TYR341, LEU345, SER339	SER339	TYR371
5,281,691	Rhamnetin	−8.8	ALA185, LEU376, LEU377, HIS193, HIS374, HIS372, TYR198, ASN368	ALA185	TYR198, ASN368
5,281,613	Diosmin	−9.0	TYR373, LEU377, HIS374, HIS372, ASN368, LEU280, VAL433, TYR371, ALA185	TYR371, HIS374	HIS372, ASN368, ALA185
5,280,647	Gossypetin	−8.8	HIS374, HIS372, HIS193, ASN368, THR198, HIS200	None	HIS374, ASN368, THR198, HIS200

The optimal chemical complex of the protein‒ligand complex, *i.e.*, PTGS2 with flavoxate, was simulated using molecular dynamics for 100 ns. Desmond’s simulated trajectories were analyzed. The root-mean-square deviation (RMSD) and root-mean-square fluctuation (RMSF) were determined via MD trajectory analysis.

Figure 101A, 1B shows the time-dependent changes in the RMSD values for the C-alpha atoms in the flavoxate-bound PTGS2 protein. The RMSD plot showed that the protein complex with flavoxate ligands was stabilized throughout the indicated time interval. Once the simulation begins, the RMSD remains between ∼1.5 and 2.5 Å for the remainder of the run. The flavoxate-bound PTGS2 protein complex remained stable over the entire time interval, according to the RMSD values. Throughout the simulation, the ligand fit-to-protein RMSD of the flavoxate-bound PTGS2 protein complex was more stable, that is, it ranged between ∼2.0 and 4.0 Å for the remainder of the run. However, some variations were observed after 60 ns but remained in the range with the least RMSD variation.

**FIGURE 10:**
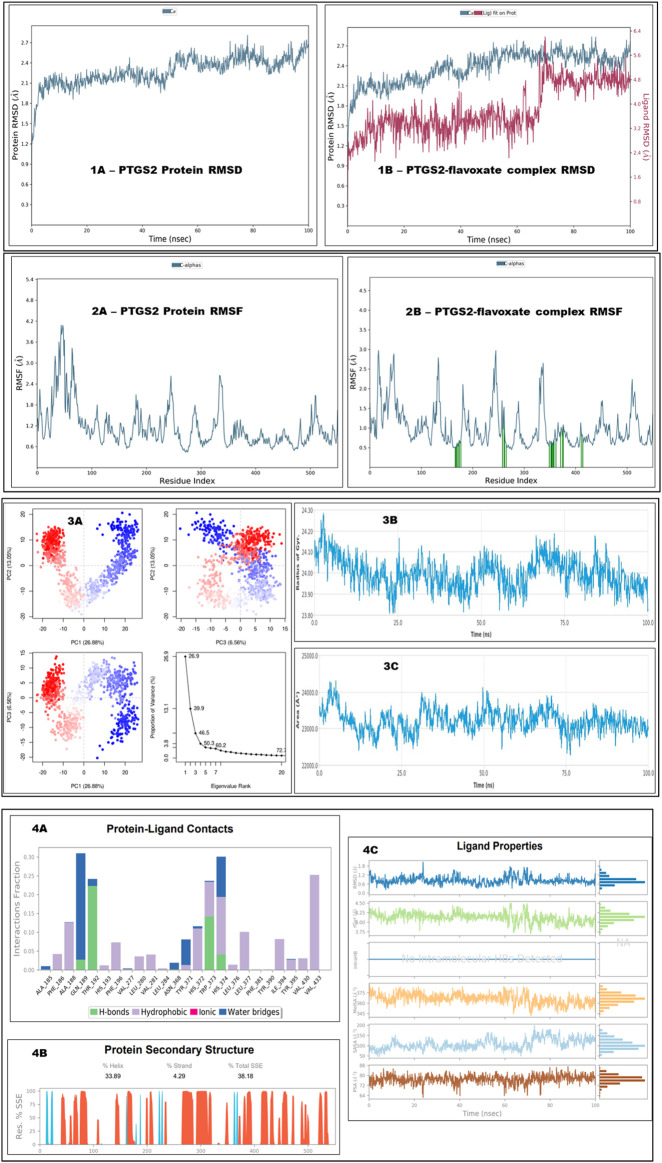
This figure shows the MD simulation analysis of unbound PTGS2 and PTGS2 bound with flavoxate at 100 ns. Section **(1A, 1B)** shows the RMSD analysis at 100 ns for both unbound protein and complex form. Section **(2A, 2B)** shows the RMSF analysis at 100 ns for both unbound protein and complex form (2B- protein residues that interact with the ligand are marked with green-colored vertical bars). Section **(3A)** shows the PCA plot analysis, **(3B)** shows radius of gyration, and 3C shows solvent accessible surface area. Section **(4A)** shows type of bonds formation during protein‒ligand contacts, section **(4B)** shows protein secondary structure elements and section **(4C)** shows ligand properties.

Figures 102A, 2B shows the RMSF values for the flavoxate-bound PTGS2 protein complex. Based on the MD trajectories, we found that the overall residues of the protein had stabilized peaks, that is, ∼2.5 Å in the N- and C-terminal zones. The stability of ligand binding to the protein was demonstrated by the low RMSF values of the binding site residues. The flavoxate-bound PTGS2 protein complex included HIS374, VAL281, LEU280, ILE394, GLN189, ALA188, TRP373, and HIS193, which exhibited a greater interaction fraction during the 100 ns analysis. Figures 103B also shows the radius of gyration and analysis of the protein‒ligand interaction area. The PCA eigenvalue was plotted against the percentage of variance, which showed some varying areas that were displayed in three separate sections. Variations in PC1, PC2, and PC3 accounted for 26.88%, 13.05%, and 6.56%, respectively (Figure 103A).

Protein–ligand interaction analysis revealed the active and dominant involvement of hydrophobic interactions with the top key amino acid residues. In addition, water bridge and hydrogen bond formations were observed (Figure 104A). Secondary structural features, such as alpha helices and beta strands, were tracked throughout the simulation (SSE). The percentage of helices was 33.89, the percentage of strands was 4.29%, and the percentage of secondary structure elements (SSEs) was 38.18% (Figure 104B). The ligand properties also summarized the different conformational evolution patterns of flavoxate throughout the simulation trajectory (from zero to 100 ns) (Figure 104C).

### 3.7 Pharmacokinetic analysis of the potential drug molecules

The detailed ADMET analysis of the six top-ranked docked candidate flavonoid compounds showed that these compounds were safe for use as drugs (Supplementary Table S4). Small molecules are generally preferred for drug development because they are easier to handle and tend to exhibit better bioavailability. The flavonoid “3-hydroxyflavone” has the lowest molecular weight (238.24), making it a favorable candidate for this purpose. LogP is a measure of the lipophilicity of a molecule and can affect its ability to cross cell membranes. Low logP values are generally preferred. Gossypetin had the lowest LogP (1.6936), indicating good hydrophilicity. The H-acceptor and H-donor parameters are related to the ability of the molecule to form hydrogen bonds. Generally, molecules with moderate numbers of H-acceptors and H-donors are preferred. Diosmin had the highest number of H-acceptors and H-donors. A higher water solubility is desirable for drug candidates because it can enhance their bioavailability. Flavoxate has relatively good water solubility. Caco2 permeability is a measure of a molecule’s ability to pass through intestinal cells. Higher values were generally preferred. Flavoxate has a high Caco2 permeability. Molecules that are not P-glycoprotein substrates or inhibitors may have advantages in terms of drug development. Neither “gossypetin” nor “rhamnetin” are substrates or inhibitors. The volume of distribution in humans can indicate the extent of the distribution of a molecule in the body. Values close to zero were preferable. “3-Hydroxyflavone” has a relatively low VDss value. The unbound fraction parameter is related to the fraction of the drug that is not bound to proteins in the bloodstream. A higher unbound fraction can enhance the efficacy of a drug. “Diosmin” has a relatively high fraction of unbound sites. The blood‒brain barrier (BBB) and central nervous system (CNS) permeability are essential for drugs to target the brain. Molecules that can cross the BBB and exhibit CNS permeability are preferable. None of the molecules in the dataset exhibited strong BBB or CNS permeability. A lower total clearance suggested a longer duration of action of the drug. “7,8-Dihydroxyflavone” had the lowest total clearance value. Molecules with no AMES toxicity are preferred because they are less likely to be mutagenic. None of the tested molecules exhibited AMES toxicity. Molecules that do not inhibit the hERG channel are preferred because hERG inhibition can lead to cardiac side effects. Maximum tolerated dose and toxicity values, as well as acute and chronic toxicity data, should be evaluated to ensure the safety of the molecule as a drug. “Flavoxate” has a relatively high “Maximum Tolerated Dose (humans)". Overall, based on the properties evaluated, “flavoxate” appears to have several favorable characteristics for further analysis as an optimal candidate compound (Supplementary Table S4).

## 4 Discussion

PTB remains a significant global public health concern, necessitating evidence-based strategies, particularly in low-resource environments, to prevent premature birth and mitigate its impact on preterm neonates (Newnham et al., 2017). Clinically, PTB may be considered an adverse pregnancy outcome (for which a fetus is unable to fulfill its pregnancy outcome *in utero*) (Medley et al., 2018). Specifically, pregnancy-related pathologies, including IUGR and PE, are facilitated by trophoblasts’ inability to remodel spiral arteries during placentation, which results in an overabundance of reactive oxygen species (ROS). Endothelial dysfunction is intensified by subsequent peroxidation, which also alters the release of proangiogenic and vasodilation molecules and damages the endothelium (Burton and Jauniaux, 2018; Joo et al., 2021). IUGR and PE, which are associated with distinct maternal health conditions, are common placental abnormalities and contribute to the occurrence of PTB (Manokhina et al., 2017). The genetic contribution to establishing a molecular biomarker to investigate the onset of PTB related to IUGR and PE is a pressing need to identify precise intervention targets rooted in genetic pathways that could be helpful in exploring better treatment strategies to improve maternal and child health. However, the molecular mechanism underlying the association between PE and IUGR has not been determined. Therefore, we analyzed PTB gene sequencing data from females with PE and those with PE + IUGR to identify key gene(s), their molecular pathways, and potential therapeutic interactions.

PE, IUGR, and PTB have complex biochemical mechanism(s) that can have genetic influence as the main component, among other contributing factors. To date, several genetic factors and pathways that may be involved in PTB-associated PE and IUGR have been identified (Steinthorsdottir et al., 2020; Wadon et al., 2020; Cai et al., 2022). However, the exact genetic mechanisms underlying these conditions have not yet been fully elucidated. In this study, we analyzed the genes associated with PTB that are differentially expressed between the placenta of patients with PE and that of patients with both PE and PE + IUGR (Awamleh et al., 2019; Yang et al., 2020). The ratio of overexpressed genes was greater in the case of PE than in the case of under expressed genes, while the opposite was true for PE + IUGR, where the number of downregulated genes was greater than that of overexpressed genes.

PE is a hypertensive disorder that occurs after 20 weeks of gestation and can cause complications in both mothers and babies (Steinthorsdottir et al., 2020; Yang et al., 2020). Genetic variations, including those in blood pressure regulation, the immune response, and vascular function, are believed to contribute to this condition. Several studies have suggested that variations in the genes related to angiogenesis (formation of new blood vessels) and oxidative stress may contribute to the development of PE (Anto et al., 2018). IUGR is a condition in which a baby fails to reach its expected growth potential owing to genetic, placental, maternal, or environmental factors (Cai et al., 2022). In the present study, while selectively focusing on the upregulated genes from the PE + IUGR dataset, we found that these genes were involved in pathways such as the apelin signaling pathway according to the KEGG ID; hsa04371 and 5 genes were involved in the total enrichment of the pathway. Initially, apelin signaling was recognized for its pivotal role in cardiovascular processes (Szokodi et al., 2002). In this context, apelin serves as a robust positive inotropic agent that effectively modulates heart contractility (Szokodi et al., 2002). Furthermore, apelin functions as an angiogenic factor that actively facilitates the proliferation and migration of endothelial cells (Kasai et al., 2004; Chaves-Almagro et al., 2015), thereby supporting the expansion and refinement of the vascular network. The consequences of the upregulated apelin pathway in the context of PE and IUGR, an augmented apelin signaling pathway, may have multifaceted effects on the cardiovascular system (Wysocka et al., 2018). It could affect heart contractility, vascular function, and angiogenesis, possibly exacerbating the cardiovascular aspects associated with PE + IUGR, characterized by hypertension and impaired vascular function (Przewlocka-Kosmala et al., 2011; Antushevich and Wójcik, 2018; Wysocka et al., 2018). It has also been reported that women who develop PE or have fetal growth restriction leading to PTB during pregnancy are at a higher risk of cardiovascular disease later in life (Giguère et al., 2012; Hermes et al., 2012).

Several genetic factors related to inflammation, hormonal regulation, and uterine functions have been implicated in PTB (Couceiro et al., 2021). Variations in the genes encoding proteins involved in the inflammatory response, such as cytokines and chemokines, can affect the risk of PTB (Green and Arck, 2020; Couceiro et al., 2021). Genetic factors related to protein encoding, which may be associated with the structure and function of the cervix and uterus, as well as those involved in the regulation of uterine contractions, can also contribute to PTB risk (Green and Arck, 2020; Couceiro et al., 2021; Cai et al., 2022). Similarly, we analyzed the PPI networks of the extracted genes, which can be helpful in providing significant molecular insights into the molecular basis of diseases. Identifying protein interactions involved in the above pathological issues may result in the investigation of molecular targets (overexpression), which can ultimately uncover key disease mechanisms and potential therapeutic targets. Sequencing data obtained from PE and PE + IUGR, which target specific proteins or interactions within the network, can also aid in the development of prominent drug targets that can modulate these pathological interactions. We discovered the key proteins (hub proteins) that were the most significant gene targets in the network related to PE and PE + IUGR. PTGS2, ENG, KIT, MME, CGA, GAPDH, GPX3, and P4HA1 are central to the network of preeclampsia-related DEGs and are associated with angiogenesis, inflammation, and hCG hormone maintenance (Liu et al., 2019; Yang et al., 2020). The network of the PE + IUGR gene set included 9 hub genes, namely, PTGS2, FGF7, FGF10, IL10, SPP1, MPO, THBS1, CYBB and PF4, related to fibroblast growth factors, inflammation, chemotaxis and phagocytosis (Awamleh et al., 2019). Our present findings revealed that the identification of hub genes confirmed that gene(s) overexpression, specifically in PTB-related pathologies, is particularly related to variations in blood pressure regulation, the immune response, and vascular function (Turbeville and Sasser, 2020; Wadon et al., 2020; Couceiro et al., 2021).

The PTGS2 (prostaglandin-endoperoxide synthase 2) gene was the most common hub gene found under both conditions (both PE and PEIUGR). PTGS2 is encoded by the PTGS2/COX2 gene and is localized to the inner and outer nuclear membranes and the membrane of the endoplasmic reticulum at the subcellular level (Anamthathmakula and Winuthayanon, 2021). It is a 604-amino acid protein that plays a particular role in the inflammatory response (Kosaka et al., 1994). PTGS2, also known as cyclooxygenase-2 (COX-2), is an important enzyme involved in the biosynthesis of the intermediate prostaglandin H (PGH). PGH plays a critical role in repairing damage to the plasma membrane, a role substantiated by previous scientific investigations (Pathak et al., 2004). Notably, recent studies have revealed that the inhibition of COX-2 effectively hinders the process of macrophage apoptosis induced by MTB-secreted lipoproteins. Furthermore, Rand et al. showed that COX-2 can modulate the p38MAPK-PG signaling pathway, attenuating MMP-1 activity. This mechanistic insight reveals a promising therapeutic target for ameliorating inflammatory tissue damage associated with PTB (Rand et al., 2009). We reported that PTGS2 was the most common gene under both conditions; that is, PE and PE + IUGR were the commonly overexpressed hub proteins.

To hypothesize that the overexpression of PTGS2 was associated with the above PE and IUGR conditions, we selected a flavonoid compound library to investigate its *in silico* inhibitory effects and compared it with those of the different PTB drugs (ritodrine, hydroxyprogesterone caproate, retosiban, terbutaline, and allylestrenol) used to treat PTB. Flavonoids are a diverse class of naturally occurring compounds characterized by their phenolic structures. They are ubiquitously present in a wide range of biological sources, such as fruits, vegetables, grains, tree bark, roots, plant stems, and flowers, and are even found in beverages, such as tea and wine (Dias et al., 2021). Naturally, derived compounds have gained significant recognition for their remarkable health-promoting benefits. Flavonoids have emerged as indispensable constituents in various domains, including nutraceuticals, pharmaceuticals, medicinal formulations, and cosmetic products. The indispensability of these materials is primarily attributed to their multifaceted properties, including potent antioxidant, anti-inflammatory, antimutagenic, and anticarcinogenic properties (Ullah et al., 2020). Additionally, flavonoids can modulate essential cellular enzymes, further enhancing their significance in diverse applications. Similarly, the 3D structure of PTGS2 showed higher and more significant binding energies with flavoxate, diosmin, 7,8-dihydroxyflavone, gossypetin and rhamninone (in the range of −9.1 kcal/mol to −8.8 kcal/mol). These findings can serve as a basis for further exploring the role of flavonoids in the management of PTB, as the *in silico* pharmacokinetic analysis of flavonoids with good lipophilicity established flavonoids as safe natural products for further analysis. The comprehensive basis of this experiment is to develop precision, usefulness, and understanding of protein–ligand interactions, as well as to expedite attempts at rational drug design that can lead to optimization. Molecular docking involves the production of stable protein‒ligand complexes through different chemical bonding patterns, such as noncovalent interactions between the protein and ligand (prominent binding site analysis). Moreover, some of the interactions include hydrogen bonding, electrostatic interactions, van der Waals interactions, and hydrophobic interactions, which set the possible mechanism of interaction through the inhibition of protein targets by flavonoids (Patil et al., 2010).

In addition, flavoxate can efficiently cross cell membranes and is an essential characteristic of a prominent candidate drug with good absorption and distribution properties. Flavoxate is a promising drug with good absorption and distribution properties, allowing for efficient cell membrane crossing. A Caco2 permeability value of 92.498 indicates good intestinal absorption, and it does not inhibit P-glycoprotein I or II, suggesting a longer duration of action. Flavoxate is not a substrate for major cytochrome P450 enzymes and has shown low toxicity, but no acute toxicity has been observed in oral rat studies (Bertoli et al., 1976; Arcaniolo et al., 2015). It also has good permeability across the blood‒brain barrier and central nervous system, which makes it an eligible flavonoid that can be further used to establish drug designs and lead development processes to manage pathologies related to PE, IUGR and PTB.

MD simulation analysis has been reported to play a vital role in drug design by providing an understanding of the interactions and behaviors of ligands with targets (proteins). MD simulations help bridge the gap between static experimental structures and the dynamic nature of biomolecular systems (Huggins et al., 2019). Molecular dynamics (MD) simulations use basic physical principles to simulate the motion and behavior of atoms and molecules with respect to time (Hollingsworth and Dror, 2018). In addition, MD simulations provide detailed insights into the behavior and dynamics of molecules at the atomic level (Badar et al., 2022). By simulating the motion of atoms and molecules over time, researchers can observe how molecules interact, move, and change their conformation (Hollingsworth and Dror, 2018; Badar et al., 2022). This information helps us to understand the fundamental properties, mechanisms, and dynamics of molecular systems. These simulations provide a method for investigating the structure, function, and dynamics of biomolecules, including their folding, binding interactions, enzymatic reactions, and conformational changes (Hollingsworth and Dror, 2018; Huggins et al., 2019; Badar et al., 2022). We also employed this strategy on the top-ranked PTGS2-flavoxate complex and found satisfactory results, which established the role of flavonoids in the inhibition of overexpressed proteins, thereby managing PTB challenge in combination with PE and IUGR.

## 5 Conclusion

This study investigated the potential of PTGS2 inhibitors for combating PTB by analyzing the differential expression patterns between placenta with PE and placenta with PE + IUGR. We identified the hub genes associated with PE, namely, PTGS2, ENG, KIT, MME, CGA, GAPDH, GPX3, P4HA1, PTGS2, FGF7, FGF10, IL10, SPP1, MPO, THBS1, CYBB, and PF4, in the case of PE + IUGR, which is essential for managing the challenge of PTB. The results of this study also explored Flavoxate as a potential natural product for inhibiting the PTGS2 protein compared with PTB drugs. Further analysis through more comprehensive genetic screening of these conditions (PE and PE + IUGR) in relation to the present outcomes can enhance the understanding of the identification of potential targets, as well as devise prevention and treatment strategies for PTB.

## Data Availability

The datasets presented in this study can be found in online repositories. The names of the repository/repositories and accession number(s) can be found in the article/Supplementary Material.
